# Data mining of enzymes using specific peptides

**DOI:** 10.1186/1471-2105-10-446

**Published:** 2009-12-24

**Authors:** Uri Weingart, Yair Lavi, David Horn

**Affiliations:** 1 School of Physics and Astronomy, Tel Aviv University, Tel Aviv 69978, Israel

## Abstract

**Background:**

Predicting the function of a protein from its sequence is a long-standing challenge of bioinformatic research, typically addressed using either sequence-similarity or sequence-motifs. We employ the novel motif method that consists of Specific Peptides (SPs) that are unique to specific branches of the Enzyme Commission (EC) functional classification. We devise the Data Mining of Enzymes (DME) methodology that allows for searching SPs on arbitrary proteins, determining from its sequence whether a protein is an enzyme and what the enzyme's EC classification is.

**Results:**

We extract novel SP sets from Swiss-Prot enzyme data. Using a training set of July 2006, and test sets of July 2008, we find that the predictive power of SPs, both for true-positives (enzymes) and true-negatives (non-enzymes), depends on the coverage length of all SP matches (the number of amino-acids matched on the protein sequence). DME is quite different from BLAST. Comparing the two on an enzyme test set of July 2008, we find that DME has lower recall. On the other hand, DME can provide predictions for proteins regarded by BLAST as having low homologies with known enzymes, thus supplying complementary information. We test our method on a set of proteins belonging to 10 bacteria, dated July 2008, establishing the usefulness of the coverage-length cutoff to determine true-negatives. Moreover, sifting through our predictions we find that some of them have been substantiated by Swiss-Prot annotations by July 2009. Finally we extract, for production purposes, a novel SP set trained on all Swiss-Prot enzymes as of July 2009. This new set increases considerably the recall of DME. The new SP set is being applied to three metagenomes: Sargasso Sea with over 1,000,000 proteins, producing predictions of over 220,000 enzymes, and two human gut metagenomes. The outcome of these analyses can be characterized by the enzymatic profile of the metagenomes, describing the relative numbers of enzymes observed for different EC categories.

**Conclusions:**

Employing SPs for predicting enzymatic activity of proteins works well once one utilizes coverage-length criteria. In our analysis, L ≥ 7 has led to highly accurate results.

## Background

Recently there has been a rapid growth in the number of putative proteins derivable from new genomic and metagenomic data [1]. The extended use of environmental shotgun sequencing to study diverse microbial systems has made metagenomics a vastly growing field leading to a flux of data, calling for development and application of new tools that allow its investigation [2]. Conventional tools for predicting the function of a protein from its sequence are based on sequence-similarity [3] or sequence-motifs [4,5]. Here we outline a relatively simple and straight-forward method that is applicable to large numbers of sequences. Its purpose is finding whether each protein in the data is an enzyme and, if so, what its EC classification is. This Data Mining of Enzymes (DME) is based on the Specific Peptide (SP) method of [6], and is carried out by comparing the sequences of all proteins with a list of all SPs and looking for matches of the latter in the data.

SPs are strings of amino-acids, extracted from enzyme sequences using the motif extraction algorithm MEX [7]. They are selected for their specificity to levels of the Enzyme Commission (EC) 4-level functional hierarchy. We have updated the SP set of [6] by extracting it from all Swiss-Prot enzymes of July 1st, 2006. More details are provided in Methods.

Using SPs for prediction of enzymatic function needs some further decisions as to what to do if various SP hits on the same protein have EC assignments that are not consistent with one another. Moreover, one should decide when a single SP hit is sufficient to make a prediction. The methodology developed here relies on coverage length (overall number of amino-acids) of consistent SP hits. This is further described below, when testing performance on an enzyme test set, and when discussing a ten-organism test-set that contains non-enzymatic as well as enzymatic proteins. We develop a random model for the latter to assess the effect of accidental SP matches. The resulting methodology, which we call Data Mining of Enzymes (DME), is being applied to analyze several metagenomes.

## Methods

### The new SP sets

A novel method based on sequence motifs has been proposed by [6], who have studied enzymes in the Swiss-Prot database. They have demonstrated that enzyme functions, as represented by the four-level EC hierarchy, can be deduced from the appearance of deterministic short strings of amino-acids, denoted as Specific Peptides (SPs), on these enzymes. The SPs were derived from enzyme sequence data using an unsupervised motif extraction algorithm MEX [7], and filtered by the EC so that each SP is specific to a particular EC branch, specifying the EC function that the enzyme performs. Thus, if an extracted motif is found to occur on enzymes belonging to only one EC number (i.e., 4th level of the EC hierarchy), this peptide will be declared to be an SP labeled with this EC number. If, however, the motif occurs on several EC numbers, all of which share the same 3rd-level hierarchy (i.e. the first three digits of their EC numbers are the same), the motif is declared as an SP with labeling at the third level of EC hierarchy, etc. The SPs of [6] comprise on average 8.4 amino-acids (SD 4.5), and were shown to compete favorably with a Smith-Waterman based SVM classifier. Usage of the SP methodology is demonstrated by our web-tool http://adios.tau.ac.il/DME. Given the sequence of an enzyme, this tool searches through the set of all SPs and finds which of them coincide with substrings of the sequence, indicating where they lie, what is the EC assignment associated with each SP, and provides the EC predicted by the DME method for the protein that is being queried.

Kunik et al [6] have investigated 50,698 enzyme sequences of the 48.3 Swiss-Prot release of October 2005. We have used the same methodology and applied it to all enzymes in the Swiss-Prot/Enzyme records of July 1st, 2006. The number of enzymes that have a single EC assignment is 89,854. Applying MEX and filtering it by EC levels in the same way as [6], we have obtained 87,017 SPs. This new 1^st ^list of SPs serves as the basis for developing and analyzing our methodology.

In making the prediction of an EC number (i.e. 4^th ^level of the EC hierarchy) based on one SP match, or several SP matches that have the same EC number assignment, we require that the total number of amino-acids of the protein matched with these SPs be at least seven. We refer to this number as the coverage-length L. If L at level 4, L4, is less than 7, we check for SP hits that are consistent at level 3 of the EC hierarchy, i.e. have identical first three digits in their assignments. Once again, a prediction is made if L at level 3, L3, is at least 7. In principle, the threshold of L at every EC level can be viewed as a parameter of our method. Reducing L increases recall at the expense of lowering precision, as will be discussed below.

Test data were downloaded from Swiss-Prot Release 56 on July 1st, 2008. We consider two types of test sets. The "Enzyme Test Set" consists of all enzymes integrated into Swiss-Prot between July 1st of 2006 and 2008. The "10 Organisms Test Set" consists of proteins of E. coli and 9 other bacteria (see Additional file 1, Table S1 for details) containing enzymes from the same period of 2006 to 2008, and all other proteins incorporated into Swiss-Prot by July 1st, 2008. A summary of all the relevant data is displayed in Table 1. It includes also information about precision and recall (for definitions see below) that will be further discussed in the first Results section. These values are obtained by determining 3rd level EC assignments, using coverage-length of L3 ≥ 7. Precision values of 100% on the training sets are of course trivial results of specificity.

**Table 1:** Compilation of training and test datasets.

Dataset	Selection Criteria from Swiss-Prot	Number of Proteins (and SPs)	Precision	Recall
Training set #1	Single EC annotation and Date-Integrated before 7/1/2006	89,854 (#SPs = 87,017)	100%	85%
"Enzyme Test Set"	EC annotation and Date-Integrated between 7/1/2006 and 7/1/2008	24,443	98%	70%
"Ten Organism Test-Set"	EC annotation and Date-Integrated between 7/1/2006 and 7/1/2008 and all non-enzymes before 7/1/2008	4,509	98%	76%
Training set #2	Single EC annotation and Date-Integrated before 7/27/2009	201,169 (#SPs = 312,465)	100%	94%

54% of the proteins in the 1st training set carry Swiss-Prot annotations of 'active site', 'binding site' or 'metal binding site' at specific locations of single amino-acids. SPs cover these functionally important sites significantly more than other loci on proteins, thus indicating biological significance of SPs (for an extensive discussion see [8], in particular Table 1 there). SP matches that overlap such sites are compiled, and the corresponding SPs are denoted as Annotated SPs (ASPs). We have thus compiled a list of 6,078 ASPs. All appear at least four times in the training set, and the location of the annotation is consistent in the different appearances. Most ASPs carry single annotations (1,900 active sites, 1932 binding sites and 1,819 metal binding sites), 418 ASPs carry two annotations and 3 ASPs carry all three annotations.

A second set of SPs is extracted from Swiss-Prot data dated July 27th, 2009. This training set, consisting of all singly annotated enzymes, contains 201,169 proteins. It has led to 312,465 SPs. Their length distribution is presented in Additional file 2, Figure S1. This set includes 285,485 SPs with labels corresponding to EC levels 3 and 4 (containing 257,598 SPs of length ≥ 7). Only SPs with EC labels at levels 3 and 4 are relevant for the assignment of EC level-3 annotations to proteins, and hence for the calculation of recall included in Table 1. It should be emphasized that only 191,275 of the Swiss-Prot annotated enzymes in the training set carry EC annotations at levels 3 and 4. They are the ones on which the EC predictions at level 3 are tested, leading to the recall result of 94%. The 2nd SP set is being used for the analysis of metagenomic data and is incorporated in our web-tool at http://adios.tau.ac.il/DME.

### Estimate of accidental SP matches

Proteins that do not possess enzymatic functions may still have a substring that matches an SP. Such SP matches will be called 'accidentals'. Their occurrences can be modeled by SP hits on random protein sequences. Such random sequences are generated from real data by scrambling the order of the amino-acids in every protein, conserving only first-order statistics. 3 such sets were produced in order to measure the expected random hits. Estimates of the probabilities of accidental occurrences of SPs are derived below for the 10 organism test-set and for Sargasso Sea data.

### Recall-precision analysis of EC annotations in enzymes

Comparing the results of our method with an expert-method (such as Swiss-Prot) we face three possible situations when dealing with a collection of enzymes: P|P where the model prediction coincides with that of the expert, P|DP where the expert provides a different EC assignment, and NP|P where the model provides no prediction for enzymes whose EC assignments are given by the expert. Accordingly we define the following measures in terms of number of occurrences:

This is a generalization of the common terms used in binary classification problems where P|P, P|DP and NP|P are replaced by true-positive, false-positive and false-negative correspondingly.

### Recall-precision analysis of EC annotations in proteins

Extending the previous analysis to a collection of proteins we have to add two more possibilities: P|NP, where the new method has an EC prediction whereas the expert does not have one, and NP|NP where both do not have any EC assignment. Whereas the latter corresponds to true-negative in a binary classifier, the former, P|NP could be added to P|DP as 'false-positive'. Since, however, there are many cases where the absence of an EC assignment does not imply that the protein in question is not an enzyme, we opt to define a new measure, putative novelty ratio, as the fraction of such P|NP out of all the predictions of the model:

Other measures one can define are

They are the analogs of

and

in conventional binary classifications.

## Results

### Analysis of the Methodology

#### Analysis of the Enzyme Test Set using the 1^st ^SP set

In making the prediction of an EC number (i.e. 4th level of the EC hierarchy) based on one SP match, or several SP matches that have the same EC number assignments, we require that the total number of amino-acids of the protein matched with these SPs be at least seven. We refer to this number as the coverage-length L. In principle, the threshold of L at every EC level can be viewed as a parameter of our method. Reducing L increases recall at the expense of lowering precision. This is exemplified in Table 2, where we analyze our enzyme test set and show precision and recall at 3rd EC level as function of the L3 threshold.

**Table 2:** Variation of precision and recall of DME (based on the 1^st ^SP set) on the enzyme test-set as function of the L3 threshold.

L3 threshold	precision	Recall
5	95.1%	72.4%
6	95.8%	72.3%
7	98.4%	70.0%
8	99.4%	67.1%
9	99.5%	66.2%
10	99.5%	65.4%
11	99.5%	65.0%
12	99.6%	64.8%
13	99.6%	63.9%

Although precision turns out to be quite high, even for low L3 values, recall is low when compared to what BLAST [9] can achieve on this test-set. Using the most significant outcome of a BLAST search against the 1st training set as its prediction, and limiting the most significant e-value to stay below e-05, we find BLAST precision of 98% and recall of 95%, to be compared with DME values of 98.4% and 70% when setting L3 ≥ 7. Thus while precision is similar, DME loses on recall. There is no direct relation between DME and BLAST, although high coverage-length L values of DME go usually hand in hand with very low e-values of BLAST. Differences may occur for low L values of DME, and relatively high e-values in BLAST. We refer to Kunik et al. [6] for a discussion of such examples (see Table four there). The advantages of SPs in resolving classification problems in situations of remote homology have been discussed and exemplified by [8].

It is worthwhile pointing out that the fact that one can abide by such a small threshold value of L ≥ 7 is strongly connected to our requirement that the SP matches on the protein's sequence be exact. If one were to allow for insertions or deletions or replacements, such as the BLOSUM62 matrix [10], this would not work. Based on various trials we may state that, whereas reliance on BLOSUM works well for BLAST searches over large sequences, it ruins predictivity and specificity of SP searches even if only single amino-acid changes are allowed.

#### Analysis of the ten organism test-set

**Figure 1:**
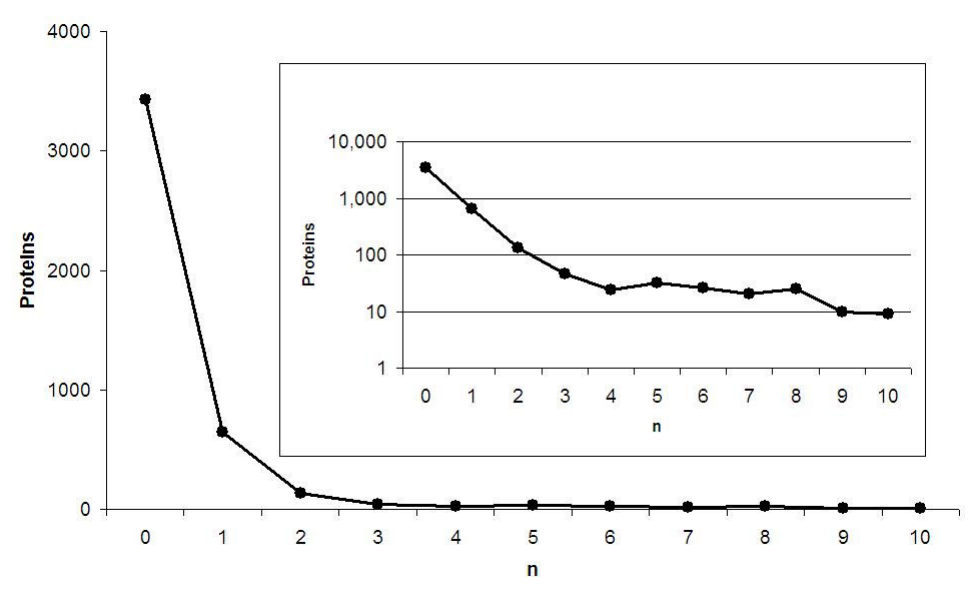
**SP hits on the ten-organism test-set**. The numbers of proteins in the ten organism test-set carrying n SP matches, for n = 0 to 10. The inset shows the same data on a semi-log scale, emphasizing the sharp exponential decrease for low n, partially reflecting the existence of erroneous SP hits.

The ten organism test-set contains 4,509 proteins of E. coli and 9 other bacteria listed in Additional file 1, Table S1. Proteins for this dataset were downloaded from Swiss-Prot on July 1^st ^2008 and include all proteins that had no EC annotation in Swiss-Prot prior to July 1^st ^2006. The intersection between the 10 organism test-set and 1^st ^training set used to build the SPs is void and allows us to develop and test the SP methodology on general proteomic data rather than on enzymes only. SP search on this dataset, using our 1^st ^set of 87,017 SPs (see Methods), leads to the results shown in Figure 1, sorted according to the number n of SP matches.

1,079 proteins have at least one SP match (or 'hit'). Some of them may be due to random hits and our task is to resolve which of the hit proteins should be recognized as enzymes and what their EC assignments should be. As before, we propose to rely on coverage length. We judge the prediction not by how many SP hits (with consistent annotations) are observed, but by L3, the number of amino-acids matched by all SP hits whose EC assignment is identical within the first three digits of the EC number. In order to have some intuition about the expected noise level, we compare in Table 3 SP hits on real data with random model results for different values of L3. Entries of L3 = 0 refer to either no SP hits, or hits by SPs that have labels with EC levels 2 and 1 but none at EC levels 3 and 4. The columns random and stdev refer to the average and standard deviation of three random sets. Noise is the ratio of random/real. All 4509 proteins of the ten-organism test-set were included in this search.

**Table 3:** Comparison of results for the ten organism test-set with those of a random model as function of coverage-length at level 3 of the EC hierarchy.

L3	Real	Random	stdev	Noise
0	3768	4150.33	18.8	
4	0	1.00	0	
5	41	41.67	1.53	1.02
6	305	256.00	19.1	0.84
7	106	54.33	1.15	0.51
8	13	3.67	2.89	0.28
9	5	0.00	0	0
10	2	0.00	0	0
11	1	0.00	0	0
12-15	25	2.00	1.73	0.08
>15	243	0	0	0

We will use L3 ≥ 7 as our threshold criterion, as in the enzyme data-set discussed in the previous section. We note that predictions based on L3 = 7 may still have a large uncertainty, however from L3 = 8 onwards random hits become very small. Our threshold criterion leads to the results displayed in Table 4, with precision = 98.4%, recall = 75.9%, accuracy = 95.1% and putative novelty = 35.2%.

**Table 4:** DME predictions vs. Swiss-Prot EC (level 3) annotations for the 10 organism Test Set.

	DME	Swiss-Prot	# proteins
A	P	P	252
B	P	DP	4
C	P	NP	139
D	NP	P	76
E	NP	NP	4,038

The interest in this exercise is twofold: to see how well our method performs on unassigned proteins, i.e. true-negatives, and how good our predictions are for putative novelties. Indeed, our accuracy turns out to be high, 95.1%, which proves that we have correct negative assignments.

Seven out of the 139 putative novelties (category C in Table 4) have been annotated by Swiss-Prot since July 2008, six out of which are at levels 3 or 4. All observations are consistent with the predictions, as shown in Table 5. Quoted here are also all coverage lengths on which the predictions were based. Note that also the one based on coverage length 7 has been validated. All this may be viewed as an indication (although not a proof) of the validity of DME predictions. The first six entries in Table 5 belong to E. coli, and the last protein belongs to Bacillus cereus.

**Table 5:** DME predictions for the ten-organism test-set are compared with recent Swiss-Prot EC assignments.

id	DME Prediction (1^st ^SP set)	L1	L2	L3	L4	Current Swiss-Prot EC annotation
P06610	1.11.1	25	22	22	0	1
P07821	3.6.3	25	25	25	0	3.6.3.34
P0A9V1	3.6.3	7	7	7	0	3.6.3
P33360	3.6.3	13	13	13	0	3.6.3
P76469	4.1.2	9	9	9	0	4.1.2.n3
P77257	3.6.3.17	14	8	8	8	3.6.3
Q81IT9	3.6.1	58	58	58	0	3.6.1

L1 to L4 are the coverage-lengths at EC levels 1 to 4 respectively

#### Classification based on Annotated SPs

It has been noted by [6] and [8] that some of the SPs can be demonstrated to play important biological roles since they carry crucial amino-acids known to serve as active sites, binding sites or metal binding sites. Such annotations are available for 54% of the enzymes in the 1^st ^Swiss-Prot training set. Selecting only SPs that carry these annotations we obtain a set of 6,078 Annotated SPs (ASPs), a mere 7% of all SPs. We have tested it on the enzyme test set. Using annotation predictions at the third level of EC we find precision 99.6% and recall 25.4%. The limited recall is due to the fact that ASPs have been derived from only 54% of the training set. Nonetheless they possess the advantage of being selected due to their demonstrated operational importance to the catalytic function. Because of their limited recall we have not used the ASPs as the primary tool for large scale analysis; however we list their properties in our web tool http://adios.tau.ac.il/DME. Any queried protein can be analyzed by this tool for SP hits and the expected DME prediction. The appearance of ASPs may serve as providing additional credence to the prediction, as well as specifying the positions of expected active or binding sites.

### Metagenomic Analysis

#### Analysis of Sargasso-Sea data

After verifying DME on the two test-sets we turn to an analysis of the 1,001,986 records in the Sargasso Sea protein data [11]. The average length of these proteins is 194 amino-acids, with SD = 109. For this analysis we employ our 2nd set of SPs, updated on July 2009. In order to reduce random hits, we have further limited our SP set to include only peptides of length 7 amino-acids or more. Using a random set of 5,000 proteins selected from these data, we generated three randomized protein sets from which we calculated the probabilities of accidental matches. The results are displayed as function of L3 in Table 6. The columns random and stdev refer to the average and standard deviation of three random sets. Noise is the ratio of random/real.

**Table 6:** Numbers of sequences with consistent SP hits (same category at level 3 of the EC hierarchy) are compared between 5000 proteins randomly chosen from Sargasso-Sea data, and a corresponding random model, as function of coverage-length.

L3	Real	Random	stdev	Noise
0	3,910	4,868	5.1	
7	235	127	5.5	0.54
8	71	6	2.1	0.08
9	40	0		0
10	27	0		0
>10	717	0		0

Similar results are obtained for L4. The results of Table 6 are slightly better than Table 3. The reason is that we have limited ourselves here to SPs of individual length 7 or more. Once again we choose L = 7 as our threshold for DME predictions. Applying DME with this threshold we obtain EC assignments at levels 3 and 4 for 220,278 proteins. All assignments are provided in Additional file 1, Tables S2-S4.

**Figure 2:**
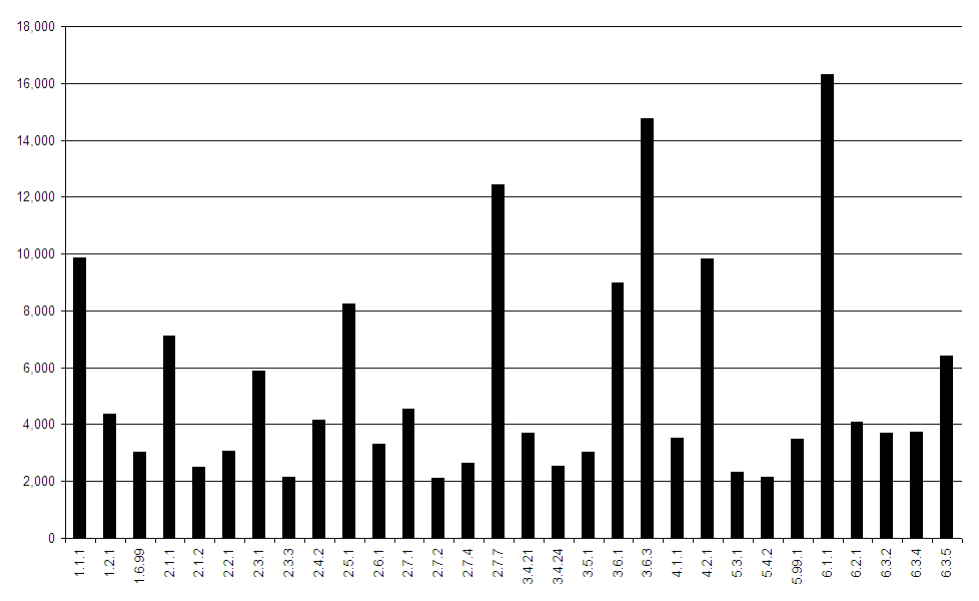
**Numbers of enzymes predicted in Sargasso-Sea data**. Numbers of enzymes predicted by DME in the Sargasso-Sea data. Shown are the thirty leading level 3 EC categories.

In Figure 2 we display a histogram of the 30 largest EC sub-subclasses (level 3) that emerge from our DME analysis. The category with the largest number of different proteins is 6.1.1, corresponding to aminoacyl-tRNA synthetases (aaRS). Since there are about 20 aaRS enzymes expected for each organism, this allows us to estimate the content of the metagenome to be of order of 800 species or so. Looking at level 4 annotations, i.e. at specific aaRS enzymes, we find that their numbers vary from 116 to 1326. These differences may be due both to different occurrences of aaRS sequences in the sample, and to different efficiencies of the SP methodology for different aaRSs. The order of magnitude of 1000 different species remains a reasonable estimate. The same order of magnitude can be derived from another source. Venter et al. [11] have provided some information about single copy proteins (Table two there) in trying to arrive at estimates of the number of species involved. One such protein is the gyrase subunit B enzyme, GyrB. The same enzyme has also been proposed by Watanabe et al. [12] for the purpose of spanning a database for identification and classification of bacteria. GyrB is one of several protein families belonging to EC 5.99.1.3 (DNA gyrase). Checking through the SPs belonging to this EC we have found a subset that is specific to GyrB only. Using this subset we estimate the number of GyrB copies in the Sargasso-Sea data to be 1344, which is close to the number of maximal fragment depth of 924 quoted in Table 2 of [11], and is in the same ball-park as the aaRS estimate.

In addition to 6.1.1 (aaRS) enzymes we observe the following leading categories: 3.6.3 (Hydrolases catalyzing transmembrane movement of substances involving ATPases), 2.7.7 (Nucleotidyl transferases), 1.1.1 (Oxidoreductases acting on the CH-OH group of donors), and 4.2.1 (Carbon-oxygen lyases).

There are several EC numbers (i.e. level 4 of the hierarchy) that are particularly abundant. They are presented in Table 7, where we list all cases that appear more than 2000 times in the data. Some of them have already been mentioned above: the DNA gyrase, and its role in estimating the number of species, and the two ECs belonging to the susubclass of 2.7.7 (Nucleotidyl transferases), playing important roles in RNA and DNA polymerases.

**Table 7:** Leading occurrences of EC-numbers in Sargasso-Sea data

EC	# proteins	Enzymatic activity
2.7.7.6	5,993	DNA-directed RNA polymerase
1.6.99.5	2,999	NADH dehydrogenase (quinone)
5.99.1.3	2,610	DNA topoisomerase (ATP-hydrolysing). DNA gyrase.
6.3.5.5	2,198	carbamoyl-phosphate synthase (glutamine-hydrolysing)
3.6.3.14	2,169	H^+^-transporting two-sector ATPase. ATP synthase.
2.7.7.7	2,083	DNA-directed DNA polymerase

All our predictions for the enzymatic annotations of the Sargasso-Sea data are presented in Additional file 1, Tables S2-S4. We wish to point out that some of the enzymes contain two or more EC assignments. Table 8 reports some of these occurrences. Included here are the most abundant observations of dual EC assignments, sorted by the numbers of proteins exhibiting the two annotations.

**Table 8:** Some examples of doubly annotated enzymes uncovered by DME in the Sargasso-Sea data.

Prediction a	Prediction b	# Proteins
3.5.4.25	4.1.99.12	27
3.6.3.44	2.7.1.130	6
1.1.1.205	1.7.1.7	6
2.7.1.25	2.7.7.4	6

The first and the last entries in Table 8 have many analogs in currently known doubly-annotated enzymes in Swiss-Prot. Checking all proteins we find that the SP hits that belong to the two different EC numbers do not overlap on the protein sequences, thus falling comfortably into the categorization of two different catalytic domains. It is interesting to note that finding multiple domains is easier with SPs than it is with BLAST: we will not miss out on a small domain of a protein that may be overshadowed by sequence similarities with a larger protein domain, and we can immediately check whether the different catalytic regions lie on disjoint sections of the protein. A full list of the doubly annotated Sargasso-Sea enzymes is presented in Additional file 1, Table S3. A further list of triple-enzymatic annotations is presented in Additional file 1, Table S4.

#### Human Gut Metagenome

Gill et al. [13] have analyzed the DNA sequences obtained from fecal DNA of two healthy adults - 'subject 7' a female aged 28 and 'subject 8' a male aged 37. We have analyzed the resulting proteins (downloaded from http://img.jgi.doe.gov/m/) with our DME method. The two proteomes of subjects 7 and 8 consist of 20,523 and 25,980 proteins correspondingly. We predict enzymatic annotations for 3,428 proteins of subject 7 and 4,102 proteins of subject 8. These numbers are relatively lower than the enzymatic content of Sargasso-Sea. Numbers of 6.1.1 enzymes are predicted to be 260 and 264 for subjects 7 and 8 respectively. Thus the number of different species contained in these samples is scaled down by two-orders of magnitude compared to the Sargasso-Sea data, which is quite reasonable given the size of the databases. Further comparisons between the three metagenomes are offered in the next section.

#### Enzymatic Profile

Trying to compare different metagenomes with each other one has obviously to resort to some normalization method. Normalizing the results of a histogram like Figure 2 by the total number of enzymes that we find, we obtain a spectrum characteristic of the genome or metagenome we study, which we will refer to as its enzymatic profile.

**Figure 3:**
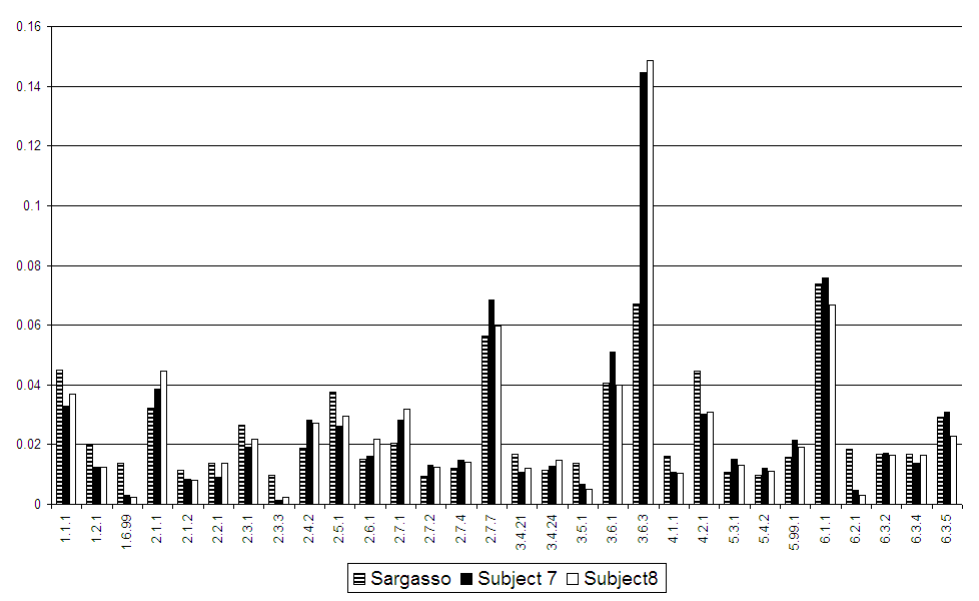
**Enzymatic profiles**. Enzymatic profiles of three metagenomes. Compared are the relative numbers of identified enzymes in the 30 leading sub-subclasses (EC level 3) of the Sargasso-Sea meatagenome with those of the gut microbiomes.

Figure 3 depicts such profiles for the examples studied in this paper, the Sargasso Sea one, and the two gut metagenomes, all based on DME predictions. Since all three are bacterial metagenomes the leading EC categories are quite similar. The identities of the leading categories have already been described in the previous section.

In spite of the obvious similarities, there exist differences among the three histograms. We use the absolute value of the difference of any two distributions as the difference measure (theoretically limited to vary between 0 and 2). Taking into account all level-3 EC predictions we obtain the distances between the different distributions presented in Table 9. As expected, the two gut metagenomes are the closest pair.

**Table 9:** Absolute values of differences between enzymatic profiles, based on the DME predicted distributions at level 3 of EC.

Metagenome	Sargasso	Subject7	Subject8
**Sargasso**	0	0.42	0.41
**Subject7**	0.42	0	0.18
**Subject8**	0.41	0.18	0

It has been emphasized by [14] and by [15] that the functional characteristics of a metagenome vary with the environment in which it is being found. Hence we expect the genetic enzymatic profiles to vary accordingly. Our exercise shows that the gross features of microbial communities may be similar, thus more attention will have to be paid to smaller details, in particular emphasizing the cases where the relative differences between EC categories are the largest. This may become a useful tool in the future.

We wish to close this section by emphasizing that the three metagenomic profiles are different from those derived from the genome of E. coli, and very different from human. The comparisons are presented in Additional file 2, Figure S2, drawn according to the top 20 categories of E. coli, and Additional file 2, Figure S3, displaying the top 20 categories of human. It is quite evident that the weights (or numbers of different genes) of different EC categories change considerably from human to E. coli to bacterial metagenomes. This implies that enzymatic profiles contain information that may be of value in future studies of novel genetic material.

## Discussion

Using SPs it seems quite straightforward to perform data-mining of enzymes. There are however several provisos: a) although a majority of enzymes carry SPs, there exists a minority that does not; hence not all enzymes are expected to be discovered in a new dataset. b) SPs were substantiated on a training set, and their generalization carries with it some error, even on a test set composed of enzymes only. Errors may be due to a) changes in the official EC classification of an enzyme, or b) real biological changes such as evolutionary loss of an active site in a protein that resembles a known enzyme but has no catalytic function, or c) random appearance of SPs on proteins that have no catalytic activity. Errors due to reclassification of EC numbers cannot be controlled in any a-priori manner. The question of functionality loss can be partially checked through searching for the absence of annotated SPs in cases where such annotations may be expected for the enzyme in question. This demonstrates the importance of detailed corroboration of each individual prediction of the large-scale method studied here. The third source of errors, due to random appearance of SPs on proteins other than enzymes, has been taken into account by limiting our predictions to consistent SP hits with minimal coverage length of 7, and specifying the L values of our predictions as a measure of their confidence.

DME is based on deterministic motifs only, i.e. strings with specific sequences of amino-acids. Comparing it with the well-known motif method of Prosite patterns [5], by using available information in Swiss-Prot, we find that the latter has precision of 97% and recall of only 47% on the Enzyme test set, thus falling short of DME predictions. When comparing DME to BLAST on the enzyme test-set we found that DME had comparable precision (98.4% vs 98%) while BLAST has much better recall (95% vs 70.0%). Note that this comparison was based on the 1^st ^SP set of July 2006.

It should be appreciated that the comparative procedure based on the Enzyme test set has some bias in favor of BLAST, because the latter serves as one of the inputs to Swiss-Prot assignments. As a result, cases of remote homology which may be captured by DME could have been missed by BLAST-based assignments, as was demonstrated by [6] and by [8]. The SP-based search has two other advantages over BLAST: it is conceptually simpler, relying only on a look-up table, and it points to specific locations on the queried protein which may be relevant to the expected catalytic function of that enzyme. Hence it may have wide practical implications for enzyme research and development.

In spite of all the precautions outlined in the first paragraph, our predictions concerning the 10 organism test-set reported in this paper, do extremely well. Moreover, note that the recall quality of SPs on their training sets increased dramatically from 85% in 2006 to 94% in 2009 (see Table 1). This means that the minority of enzymes without SP hits diminishes with time. The reason is quite clear: MEX thrives on redundancy of patterns in the data. Therefore, the more proteins of the same family there are in the database, the better MEX will perform. As these lists fill up in the Swiss-Prot database, they can be better represented by simple SP motifs. Higher recall on the training set will undoubtedly reflect itself also as higher recall on future test sets, thus suggesting that the gap between the recall of BLAST vs DME will shrink with time. Indeed, carrying out a DME analysis, based on the 2^nd ^SP set, of 19,849 enzymes that have been added to Swiss-Prot from July 28 to Sep 29, 2009, we find on this novel test set precision of 99.2% and recall of 92.4%. This is a considerable increase over the recall of 70% of the 1^st ^SP set measured on the enzyme test set (see Table 1).

A straightforward peptide characterization of protein families seemed hopeless a decade or two ago, and hence necessitated the development of more sophisticated approaches such as BLAST, to quantify sequence similarities. Our analysis demonstrates that this has changed with time (and increasing amounts of data) so that nowadays the SP approach may be regarded as a useful tool, leading to valuable information. Such information, for three metagenomic data-sets, has been presented here as an example of the power of our novel methodology.

## Conclusions

The requirement that SP occurrences on protein sequences has some minimal coverage length, e.g. L ≥ 7 amino-acids in our analyses, leads to the novel tool of DME. It is applicable to large genomic and metagenomic data, and provides a good indicator for the enzymatic classification of the queried proteins, based on a look-up table only. A web tool identifying SP (and ASP) occurrences on any queried protein sequence, and providing the EC prediction of DME, is available online at http://adios.tau.ac.il/DME.

## Authors' contributions

UW developed the DME criteria, extracted the test-sets and the 2^nd ^SP set, and performed DME analysis of genomic and metagenomic data. YL extracted the 1st SP set, and carried out preliminary analysis of the gut metagenomic data. DH conceived the study, participated in its design and coordination and drafted the manuscript. All authors read and approved the final manuscript.

## Supplementary Material

Additional file 1 **Supplementary tables**. Table S1: List of the ten organisms used as a test-set. Table S2: List of DME predicted single EC annotations of proteins in Sargasso-Sea data. Table S3: List of DME predicted double EC annotations of proteins in Sargasso-Sea data. Table S4: List of DME predicted triple EC annotations of proteins in Sargasso-Sea data. Click here for file

Additional file 2 **Supplementary figures**. Figure S1: Length histogram of the 2nd SP set. Figure S2: Comparison of enzymatic profiles based on the 20 leading categories of E. Coli. Figure S3: Comparison of enzymatic profiles based on the 20 leading categories of human. Click here for file
