# Supplementary material for: Data mining of enzymes using specific peptides
Source: BMC Bioinformatics. 2009 Dec 24;10:446. doi: 10.1186/1471-2105-10-446 (PMC2811123; doi:10.1186/1471-2105-10-446)
Supplement: Additional file 1 — Supplementary tables. Table S1: List of the ten organisms used as a test-set. Table S2: List of DME predicted single EC annotations of proteins in Sargasso-Sea data. Table S3: List of DME predicted double EC annotations of proteins in Sargasso-Sea data. Table S4: List of DME predicted triple EC annotations of proteins in Sargasso-Sea data. [file 1471-2105-10-446-S1.DOC]

Fig. S1: Length histogram of the 2^nd^ SP set.

Fig. S2: Comparison of enzymatic profiles based on the 20 leading categories of E Coli.

Fig. S3: Comparison of enzymatic profiles based on the 20 leading categories of human.
