# Supplementary material for: Data mining of enzymes using specific peptides
Source: BMC Bioinformatics. 2009 Dec 24;10:446. doi: 10.1186/1471-2105-10-446 (PMC2811123; doi:10.1186/1471-2105-10-446)
Supplement: Additional file 2 — Supplementary figures. Figure S1: Length histogram of the 2nd SP set. Figure S2: Comparison of enzymatic profiles based on the 20 leading categories of E. Coli. Figure S3: Comparison of enzymatic profiles based on the 20 leading categories of human. [file 1471-2105-10-446-S2.DOC]

**Table S2.**

**List of single DME predicted EC annotations of proteins in Sargasso-Sea data**

Download zip file from <http://adios.tau.ac.il/DME_Additional_Material/>

**Table S4**
